# Effects of Different Trunk Training Methods for Chronic Low Back Pain: A Meta-Analysis

**DOI:** 10.3390/ijerph19052863

**Published:** 2022-03-01

**Authors:** Dhananjaya Sutanto, Robin S. T. Ho, Eric T. C. Poon, Yijian Yang, Stephen H. S. Wong

**Affiliations:** 1 Department of Sports Science and Physical Education, The Chinese University of Hong Kong, Shatin, Hong Kong; damien.sutanto@link.cuhk.edu.hk (D.S.); robinho@cuhk.edu.hk (R.S.T.H.); yyang@cuhk.edu.hk (Y.Y.); 2 Department of Health and Physical Education, The Education University of Hong Kong, Taipo, Hong Kong; ericpoon@eduhk.hk

**Keywords:** low back pain, rehabilitation, exercise therapy

## Abstract

We conducted a systematic review and meta-analysis comparing motor control, isometric, and isotonic trunk training intervention for pain, disability, and re-injury risk reduction in chronic low back pain patients. The EMBASE, MEDLINE, CENTRAL, PsycINFO, SPORTDiscus, and CINAHL databases were searched from inception until 25 February 2021 for chronic low back pain intervention based on any trunk training. Outcomes include the Oswestry Disability Index (ODI) and Roland Morris Disability Questionnaire (RMDQ) for disability, the Numerical Pain Rating Scale (NPRS) for pain, and the Sorensen Test (ST) for future risk of re-injury. Isometric training was superior to the control with a mean difference (MD) = −1.66, 95% confidence interval (CI) [−2.30, −1.01] in pain reduction; MD = −7.94, 95% CI [−10.29, −5.59] in ODI; MD = −3.21, 95% CI [−4.83, −1.60] in RMDQ; and MD = 56.35 s, 95% CI [51.81 s, 60.90 s] in ST. Motor control was superior to the control with a MD = −2.44, 95% CI [−3.10, −1.79] in NPRS; MD = −8.32, 95% CI [−13.43, −3.22] in ODI; and MD = −3.58, 95% CI [−5.13, −2.03] in RMDQ. Isometric and motor control methods can effectively reduce pain and disability, with the isometric method reducing re-injury risk.

## 1. Introduction

Most people experience low back pain within their lifetime [1,2], with mild to severe symptoms [3]. Physicians can identify the cause of pain 10% of the time, often giving a non-specific diagnosis [1]. Up to one-third of non-specific low back pain patients develop chronic symptoms persisting over three months [4], with a global prevalence of around 25% of the working-age population [4,5]. Trunk muscle training is often prescribed for the treatment of chronic low back pain (CLBP). Trunk muscle tension increases the spine’s ability to remain in a neutral pose under load [6] whereas spine posture and movement away from a neutral pose under load can increase the risk of spine tissue damage [7,8,9]. There is considerable trunk muscle training with no explicit clinical guidelines on the types considered effective. With CLBP significantly increasing the risk of co-morbidities including musculoskeletal, neuropathic, and psychological issues [10], we need to better understand the effectiveness of different trunk muscle training methods.

Trunk training comes in many forms [11,12,13], and most can be classified based on the biomechanical properties and training focus as:

1. Isometric (IM) training: loading the spine while the trunk muscles contract to maintain the spine in a neutral position [14]. Plank, bird-dog, and side bridge are some isometric trunk training examples.
2. Isotonic (IT) training: moving the lumbar spine through a range of motion while under load, both eccentrically and concentrically [13,15]. Sit-up and back extension focused on segmental spine movement are examples of isotonic trunk training.
3. Motor control (MC) training: isolated activation of the deep trunk musculature targeting the transverse abdominis, lumbar multifidus, diaphragm, and pelvic floor [12,16]. Some examples of this include focusing on abdominal drawing-in manoeuvre or abdominal hollowing in isolation at different positions.

Similar exercises with different training methods result in different muscle activation [17]. Altered trunk muscle activation patterns may cause CLBP, hence the MC method prioritises isolated deep activation [12]. Some types of CLBP injury can be exacerbated by a full range of motion [9] or movement under compression [8,18], hence the IM method focuses on training trunk muscle endurance while minimising spine loading [19].

Existing primary studies comparing different types of trunk training have resulted in inconsistent findings. MC could be more [20] or less effective [21] than IM in pain reduction based on the Numerical Pain Rating Scale (NPRS); more [22] or less effective [23] than IT in pain reduction; or just as [24] or more [25] effective than the control group in disability reduction based on the Roland Morris Disability Questionnaire (RMDQ). In some cases, combining different training methods also yielded inconclusive results [26,27]. Recent meta-analysis either did not specify details on the measured outcome used for disability [28], or combined the RMDQ and Oswestry Disability Index (ODI) measurements into a single outcome [27]. RMDQ and ODI have different sensitivity depending on the patient’s disability level [29], and combining them may not be appropriate. There is a lack of evidence on the comparative effectiveness of different trunk training methods in pain and disability reduction, along with the future risk of CLBP re-injury.

This study aimed to evaluate and synthesise the comparative effectiveness of MC, IM, and IT trunk training methods using a meta-analysis based on validated outcomes. This novel approach would provide clinical practitioners with more specific guidelines on CLBP training prescription. Subgroup analysis was performed to compare the effect of training duration and patient age on different trunk training methods. The subgroup analysis can provide more insights on how training duration and patient age can affect trunk training effectiveness.

## 2. Materials and Methods

### 2.1. Experimental Approach to the Problem

This study was in accordance with the Preferred Reporting Items for Systematic Reviews and Meta-Analyses (PRISMA) guidelines on systematic reviews and meta-analyses [30]. The protocol was registered on the International Prospective Register of Systematic Reviews (PROSPERO; Registration No. CRD42020168972).

Electronic databases (EMBASE, MEDLINE, PsycINFO, CENTRAL, CINAHL, and SPORTDiscus) were searched from inception until 25 February 2021, and peer-reviewed. No language restriction was applied, with a complete search strategy based on a past Cochrane review [31] available in Appendix A. Non-English journal articles were translated into English using Google Translate before data extraction. Data extracted from each study included subject demographics, inclusion and exclusion criteria, intervention details, and outcome measures.

### 2.2. Subjects

This study focused on the working-age population (19–60 years) as a significant percentage of this population experience CLBP [5,32]. The 19–25 year old population group may have different causes of CLBP and respond differently to training treatment compared to the 50–60 year old population group. Due to the non-specific nature of the CLBP diagnosis given to the 19–60 year population group, recent meta-analyses on CLBP intervention [26,27,28] have focused on this population as a whole. Many intervention studies have either used a similar age range [21,23] or do not specify any age range [33,34] in their subject recruitment criteria. CLBP is defined as persistent low back pain for at least 12 weeks [3,35] or as diagnosed by a clinician. Studies on patients with osteoarthritis, cancer, or cardiovascular disease such as claudication were excluded as their underlying condition may cause CLBP [36,37,38]. Randomised controlled trials (RCT) with patients that had undergone major spinal surgery were excluded as surgery may change the muscle, fascia, or neural structure around the lumbar area [39,40].

### 2.3. Procedures

Interventions compared for this study were trunk training that could be classified as IM, IT, or MC. Training that could not be exclusively classified as either was excluded [41,42]. Interventions combining trunk training with aerobic or limb strength training exceeding 15 min were excluded as aerobics and limb strength training may reduce the pain and disability of CLBP patients [28,43].

The included RCTs had to contain either a control or a different trunk training group as a comparator in the pre- and post-intervention. The control included passive interventions considered ineffective for CLBP, placebo intervention, or simple advice such as maintaining active daily living or exercise avoidance. Passive interventions included transcutaneous electrical nerve stimulation (TENS) [44], ultrasound [45], and patient education only [46]. Placebo intervention included detuned ultrasound, TENS, and sham massage. Home exercise prescriptions were not considered as a valid control because patient training adherence may differ and cause high measurement variability. Flexibility and mobility are not significant predictors of future CLBP [47] and have no significant effect on pain and disability reduction [28], hence brief warm-up stretching and mobility exercises on the intervention or control were acceptable.

Outcomes can be classified as Patient-Reported Outcome Measures (PROM) or Patient Performance Test (PPT) based on the COSMIN guidelines for CLBP. A recent systematic review concluded that ST has high test–retest, intra-rater, and inter-rater reliability for PPT [48]. ST is inversely correlated to CLBP risk across the study population of interest [49,50] and was chosen as the PPT for the meta-analysis.

PROMs are outcomes based on the participant’s subjective responses. A recent Delphi study concluded that NPRS, ODI, and RMDQ are the most widely accepted PROMs for CLBP intervention [51]. This review only included clinical intervention studies excluding cohort studies, case studies, commentaries, and editorials. Patients scoring 61% or above on the ODI were considered to have a crippling disability [52] and should be receiving positive intervention instead of physical training [29]. Hence, studies with patients from this group were excluded from the analysis.

Current international guidelines for the treatment of CLBP do not recommend CLBP patient subgrouping [3,35,53,54]. RCTs that use specific classification to separate patients into different treatments were excluded to ensure the external validity of the meta-analysis. Article titles and abstracts identified from the search results were independently assessed by two reviewers, with the primary research data exported to Endnote X9.2 build 13,018. Relevant grey literature was searched for related trial data. Full-text articles were screened independently by both reviewers, with a third reviewer adjudicating any disagreement. Published articles with the most relevant outcomes were included in the analysis for multiple publications from a single RCT. Publication data (author, year, and origin), study design (patients and groups numbers), intervention, and outcome from included RCTs were extracted to Table A1 in Appendix B, with the primary authors being contacted for missing data.

Review Manager v5.4.1 (Cochrane Collaboration, Copenhagen, Denmark) was used for the statistical analyses. Before input in the meta-analysis, pre- and post-intervention mean differences (MD) and standard deviations (SD) from the included studies were converted to change MD and SD based on Cochrane handbook section 16.1.3.2. Significance was set for α at 5% and 95% confidence interval (CI), and all analyses used the random effect model. Pain VAS reported as a score of 0–100 was standardised to NPRS 0–10. Weight column indicated in the meta-analysis result in Review Manager indicates the effect percentage from a particular study towards the overall MD. Green squares on meta-analysis result graphically indicates the effect of each individual study while black squares indicate the overall effect of the combined studies. Heterogeneity as I2 was classified as low (~25%), moderate (~50%), and high (~75%) [55]. High heterogeneity could be due to publication bias, methodological issues, or clinical differences. Methodological issues were investigated under the risk of bias assessment. The clinical difference was investigated using subgroup analysis. Detailed significant and insignificant results are displayed in separate figures.

The Cochrane Risk of Bias 2.0 tool was used to assess the randomisation, assessment, missing outcome, measurement outcome, and reporting outcome bias [56]. Two researchers independently evaluated and resolved differences through discussion. Sensitivity analysis was conducted by removing the high risk of bias studies. Result certainty was assessed based on the result and heterogeneity change after sensitivity analysis.

The trunk training clinical trial durations ranged from one [33] to twelve months [57]. Training duration subgroup analysis could justify a longer duration training prescription for severely affected CLBP patients. Patients under 40 may have a 3.7 times higher chance of pain and disability reduction than older patients [58]. Some intervention studies recruited patients with an age range that overlapped 40 [21,34]. Patient mean age was used to group the included studies. Studies were grouped into under-40, 40–45, and over-45 years old, with the analysis only comparing the under-40 and over 45-groups. Effects of ageing on human physiology and related training adaptation is gradual and non-uniform. Some studies with participants with a mean age of 40–45 may have an overall physiology closer to those under 40, while in other studies, the overall physiology was closer to those over 45. The 40–45 age group was not analysed to remove uncertainty in their classification as more similar to those under-40 or above-45 in the subgroup analysis. Intervention studies with the participants’ mean age of different group belonging to different classification were excluded. This subgroup analysis provided an insight into the effect of patient age on CLBP training effectiveness.

## 3. Results

**Figure 1:**
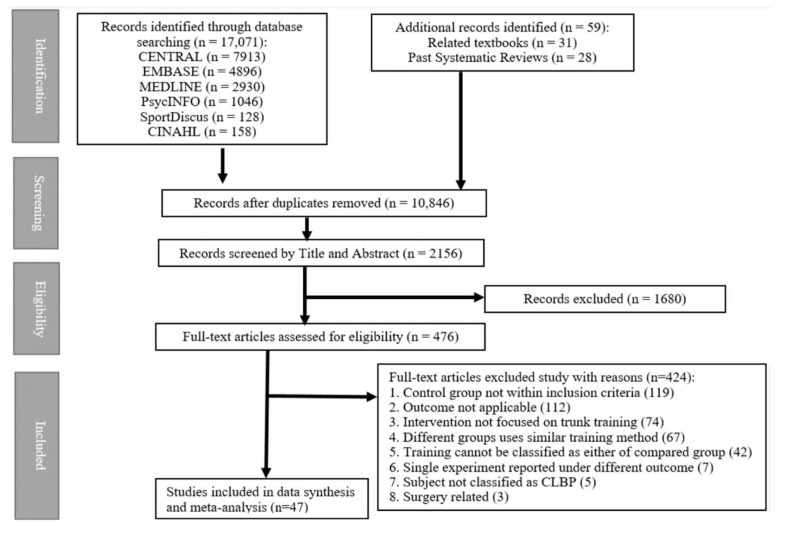
Flowchart of literature selection in the systematic reviews on trunk muscle training for chronic low back pain.

The literature search identified 10,846 citations with 10,372 citations excluded after screening the title and abstracts and full-text screening of the remaining 476 citations. One study with a related intervention and outcome was excluded as all the subjects later received surgical intervention prior to post-intervention outcome measurement [59]. Studies with non-chronic low back pain subjects were excluded [60,61] as the majority of low back pain cases resolve spontaneously within 6 weeks [62]. Forty-seven RCTs (N = 2299) were included in the meta-analysis. A PRISMA flowchart [30] of the RCT search and selection results is shown in Figure 1.

Individual study characteristics of the included RCTs are mentioned in Table A1 in Appendix B. Included RCTs were from 19 countries: 26 studies from Asia, 15 from Europe, five from America, and one from Australia. One study was in the Korean language only, while the remaining 44 were in English and another language or were written only in English. In studies with a control, seven had no details on the control intervention, five received exercise avoidance advice, four received patient education, four received passive treatment, three received a passive placebo treatment, and three were put on a waiting list. The number of patients per intervention group at baseline ranged from 5–84, with 22 of the 47 RCTs having 20–40 patients per group. Six of the included RCTs had over 40 patients per group.

Two studies recruited male patients only. Five studies did not specify their patient gender demographics. Ten studies exclusively recruited female patients and 30 studies recruited both male and female patients. Nine of the studies used four weeks as the duration for training intervention, 15 used six weeks, 14 used eight weeks, two used 10 weeks, and seven used 12 weeks. Sixteen studies used patients with a mean age below 40, nine studies used patients with a mean age 40–45, and 12 studies used patients with a mean age above 45 years old. Seven of the included studies had intervention arms belonging to different age groups and three did not have information on their recruited subject age.

**Figure 2:**
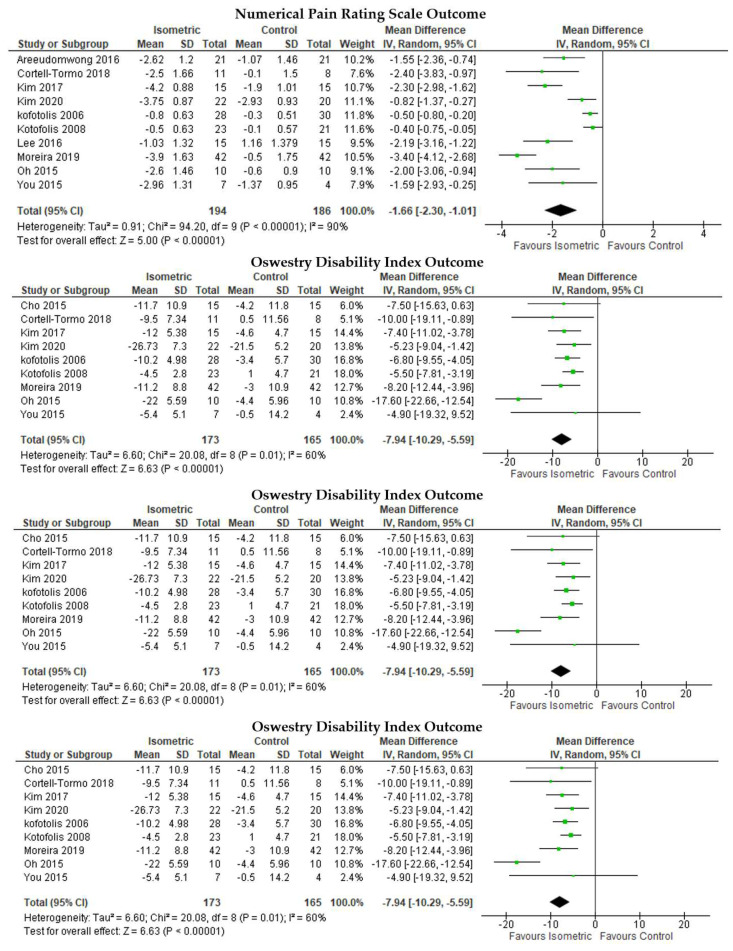
Pairwise meta-analyses on the effectiveness of isometric trunk muscle training compared to the control for chronic low back pain.

IM intervention was more effective than the control in reducing pain as measured by NPRS (Figure 2, first row), MD = −1.66, 95% CI [−2.30, −1.01], I2 = 90%, *p* < 0.01. IM was superior to the control in disability reduction as measured by ODI (Figure 2, second row), MD = −7.94, 95% CI [−10.29, −5.59], I2 = 60%, *p* < 0.01, and RMDQ (Figure 2, third row), MD = −3.21, 95% CI [−4.83, −1.60], I2 = 84%, *p* < 0.01. IM intervention increased trunk extensor endurance (Figure 2, 17^th^ row) compared to that of the control with MD = 56.35, 95% CI [51.81, 60.90], I2 = 0%, *p* < 0.01.

**Figure 3:**
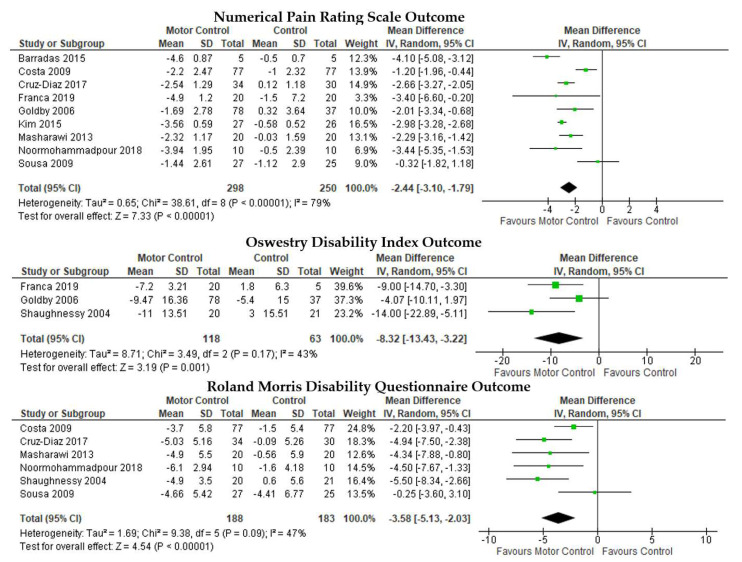
Pairwise meta-analyses on the effectiveness of motor control trunk muscle training compared to the control for chronic low back pain.

MC was more effective in reducing pain than the control as measured by the NPRS (Figure 2, first row), MD = −2.44, 95% CI [−3.10, −1.79], I2 = 79%, *p* < 0.01. MC was superior in controlling disability reduction as measured by the ODI (Figure 3, second row), MD = −8.32, 95% CI [−13.43, −3.22], I2 = 43%, *p* < 0.01 and as measured by RMDQ (Figure 3, third row), MD = −3.58, 95% CI [−5.13, −2.03], I2 = 47%, *p* < 0.01.

**Figure 4:**
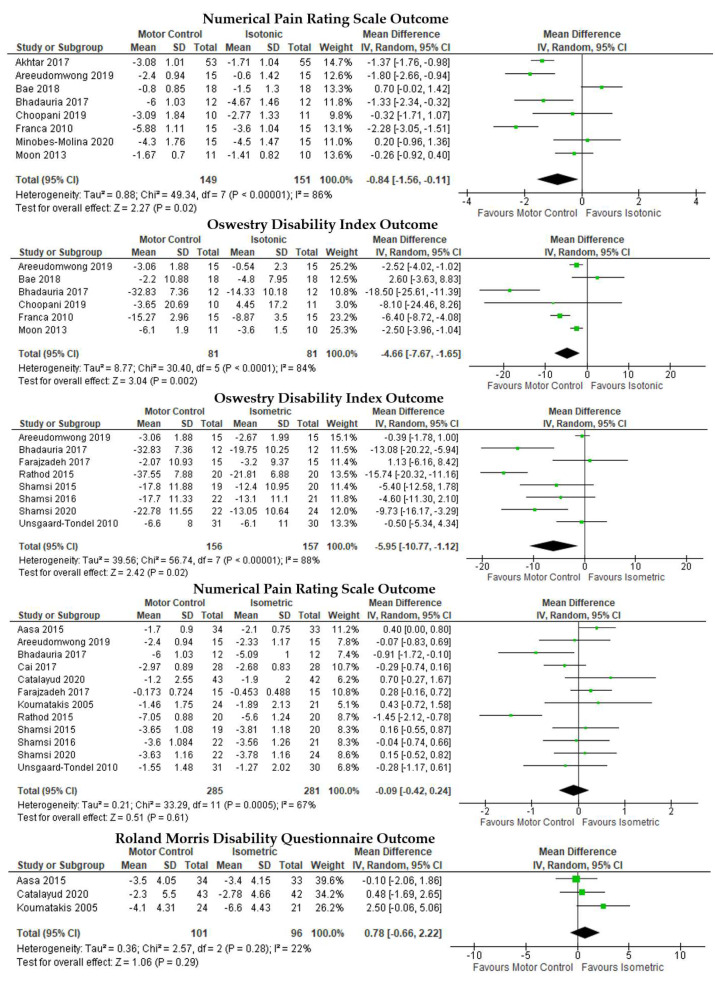
Pairwise meta-analyses on the comparative effectiveness of different trunk muscle training methods for chronic low back pain.

A pairwise meta-analysis comparing different training methods resulted in MC being superior to IT on the NPRS (Figure 4, first row), MD = −0.84, 95% CI [−1.56 to −0.11], I2 = 86%, *p* = 0.02, and ODI (Figure 4, second row), MD = −4.66, 95% CI [−7.67, −1.65], I2 = 84%, *p* < 0.01. MC was superior to IM in disability reduction based on the ODI (Figure 4, third row), MD = −5.95, 95% CI [−10.77, −1.12], I2 = 88%, *p* = 0.02. The difference was not significant as measured with the NPRS (Figure 4, fourth row), MD = −0.09, 95% CI [−0.42, 0.24], I2 = 67%, *p* = 0.61, and RMDQ (Figure 4, fifth row), MD = 0.78, 95% CI [−0.66, 2.22], I2 = 22%, *p* = 0.29.

**Figure 5:**
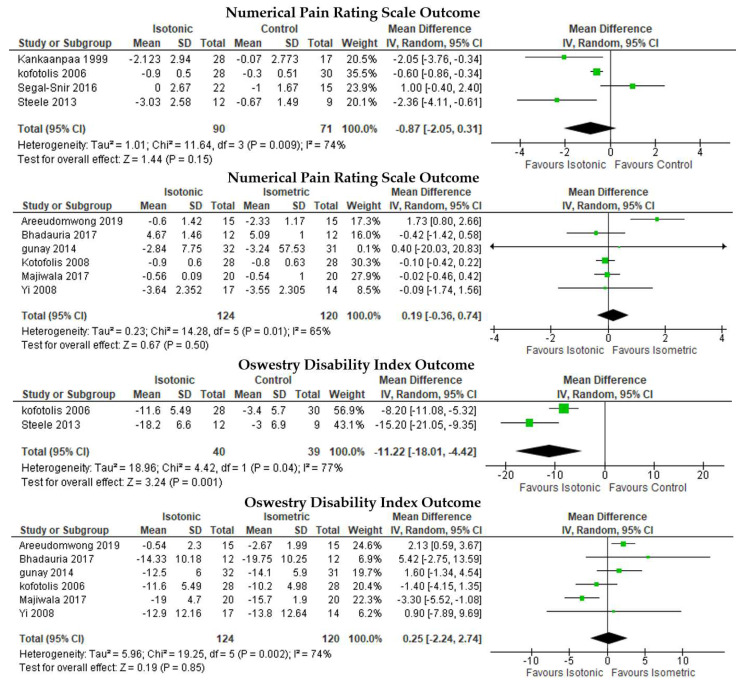

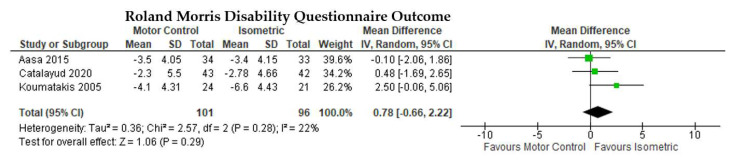
Pairwise meta-analyses with no significant results.

IT intervention did not result in significant NPRS reduction compared to the control (Figure 5, first row), with MD = −0.87, 95% CI [−2.05, 0.31], I2 = 74%, *p* = 0.15, while IM and IT intervention were not significantly different in the NPRS (Figure 5, second row) with MD = 0.19, 95% CI [−0.36, 0.74], I2 = 65%, *p* = 0.50. IT significantly reduced disability as measured with ODI compared to the control (Figure 5, third row) with MD = −11.22, 95% CI [−18.01, −4.42], I2 = 77%, *p* = 0.001. In addition, IT was not significantly different to IM in ODI reduction (Figure 5, fourth row) with MD = 0.25, 95% CI [−2.24, 2.74], I2 = 74%, *p* = 0.85. MC to IT comparison in the RMDQ outcome did not show any significant difference (Figure 5, fifth row), with MD = 0.42, 95% CI [−0.83, 1.67], I2 = 0%, *p* = 0.51. The IT to control comparison resulted in the largest disability (ODI) reduction (MD = −11.22) while MC was more effective than IT (MD = −4.66).

### 3.1. Sensitivity Analysis

**Figure 6:**
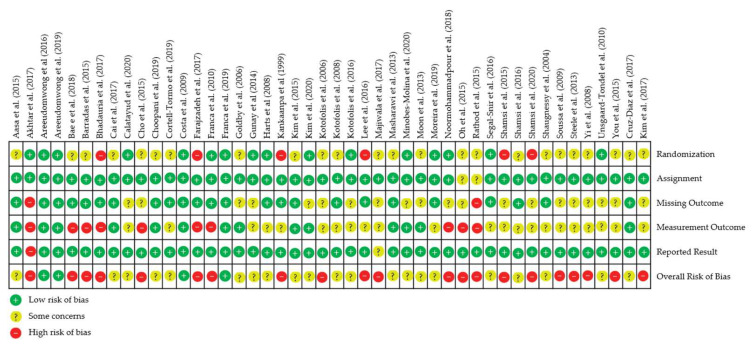
Risk of bias among the included randomised controlled trials.

Figure 6 lists the included studies along with the risk of bias in the five bias domains. Studies with low risk of bias in all five domains were judged as having low overall risk of bias. Studies with concerns in their methodology in two or less domains were judged as having some concerns in the overall risk of bias. Studies with three or more domains having methodological concerns or with high risk of bias in one of the domains were judged as having high risk in overall risk of bias. Overall, four studies had a low risk of bias, 21 had some concerns, and 21 had a high risk of bias. Ninety percent of the concerns in the randomisation bias were due to a lack of allocation concealment in the study report. Over 60% of the studies had some concerns on measurement outcome bias due to the lack of assessor blinding on group allocation, with PROMs being subjective in nature. The 21 studies with a high risk of bias were removed in the sensitivity analysis, while another study [63] was removed from the NPRS results due to missing data.

Difference in MC and IM effect on disability reduction as measured by the ODI became non-significant (Appendix C.1, sixth row), MD = −0.56, 95% CI [−1.86, 0.75], I2 = 0%, *p* = 0.40. The difference remained non-significant as measured with the NPRS (Appendix C.1, first row), MD = 0.08, 95% CI [−0.21, 0.37], I2 = 24%, *p* = 0.58. IM remained more effective than the control based on the NPRS (Appendix C.1, second row), MD = −1.55, 95% CI [−2.28, −0.83], I2 = 92%, *p* < 0.01, and ODI (Appendix C.1, seventh row), MD = −6.43, 95% CI [−7.80, −5.07]; I2 = 0%, *p* < 0.01. MC remained more effective than the control in pain reduction as measured by the NPRS (Appendix C.1, third row), MD = −2.12, 95% CI [−2.89, −1.35], I2 = 83%, *p* < 0.01. In addition, MC remained more effective in disability reduction as measured by the ODI (Appendix C.1, eighth row), MD = −10.46, 95% CI [−15.25, −5.66], I2 = 0%, *p* < 0.01, and RMDQ (Appendix C.1, eleventh row), MD = −3.44, 95% CI [−5.24, −1.63], I2 = 55%, *p* < 0.01. MC was no longer superior to IT in NPRS reduction (Appendix C.1, fourth row) with MD = −0.59, 95% CI [−1.49, 0.32], I2 = 71%, *p* = 0.20, while remaining superior in ODI reduction (Appendix C.1, ninth row), with MD = −2.53, 95% CI [−3.58, −1.49]; I2 = 0%, *p* = 0.01.

Regarding IM to IT comparison in the NPRS outcome, four of the six included studies were removed due to a high risk of bias with a sensitivity analysis showing no significant difference (Appendix C.1, fifth row), MD = 0.76, 95% CI [−1.03, 2.55], I2 = 92%, *p* = 0.40. In the IM to IT comparison in the ODI outcome, three of the six included studies had a high risk of bias according to risk of bias analysis, with no significant difference in sensitivity analysis (Appendix C.1, tenth row), MD = 0.76, 95% CI [−1.03, 2.55], I2 = 92%, *p* = 0.40.

### 3.2. Training Duration Subgroup Analysis

The difference between IM and MC effects on the NPRS (Appendix C.2, first row) outcome was insignificant for under eight weeks of intervention, MD = −0.25, 95% CI [−0.74, 0.24], I2 = 74%, *p* = 0.32, and for eight or more weeks of intervention, MD = 0.14, 95% CI [−0.28, 0.55], I2 = 47%, *p* = 0.52. Compared to a total heterogeneity of 67%, subgroup analysis resulted in lower heterogeneity in long-term intervention and higher heterogeneity in short-term intervention with no significant difference between the two groups, *p* = 0.24.

IM comparison with the control in the NPRS outcome (Appendix C.2, second row) was significant for under eight weeks, MD = −1.10, 95% CI [−1.65, −0.54], I2 = 84%, *p* < 0.01 and for eight or more weeks of training duration, MD = −2.58, 95% CI [−3.32, −1.83], I2 = 54%, *p* < 0.01. Compared to the total heterogeneity of 90%, subgroup analysis resulted in lower heterogeneity in both subgroups and greater pain reduction in longer duration interventions (*p* < 0.01).

MC comparison with the control (Appendix C.2, third row) resulted in significant NPRS reduction for under eight weeks of intervention, MD = −2.51, 95% CI [−4.12, −0.89], I2 = 91%, *p* < 0.01 and for eight or more weeks of intervention, MD = −2.47, 95% CI [−3.15, −1.79], I2 = 64%, *p* < 0.01. Compared to a total heterogeneity of 82%, subgroup analysis resulted in lower heterogeneity in long-term intervention and higher heterogeneity in short-term intervention with no significant difference between the two groups (*p* = 0.86).

IM comparison with the control in ODI outcome (Appendix C.2, fourth row) was significant for a training duration of under eight weeks, MD = −6.17, 95% CI [−7.61, −4.74], I2 = 0%, *p* < 0.01 and for eight or more weeks, MD = −12.07, 95% CI [−18.72, −5.41], I2 = 75%, *p* < 0.01. Compared to a total heterogeneity of 60%, subgroup analysis resulted in lower heterogeneity in shorter duration while increasing heterogeneity in longer duration, with no significant difference between the two groups (*p* = 0.09).

### 3.3. Age Subgroup Analysis

Subgroup analysis on IM intervention indicated that IM was effective in pain (NPRS) reduction in all age groups (Appendix C.3, first row). Patients under 40 years experienced greater pain reduction, MD = −1.99, 95% CI [−2.44, −1.53], I2 = 0%, *p* < 0.01, compared to patients over 45 years of age, MD = −1.32, 95% CI [−2.46, −0.18], I2 = 73%, *p* = 0.02. Heterogeneity of both groups was lower than the total heterogeneity of I2 = 84% with a significant difference within the groups (*p* = 0.01). IM intervention was also effective in disability reduction (ODI) among all age groups (Appendix C.3, third row). Patients under 40 years experienced similar disability reduction, MD = −7.61, 95% CI [−10.88, −4.33], I2 = 0%, *p* < 0.01, compared with that of patients over 45 years of age, MD = −10.16, 95% CI [−18.66, −1.66], I2 = 87%, *p* = 0.02. The heterogeneity of the under 40 group was lower, I2 = 0%, than the total heterogeneity of I2 = 65% with the highest heterogeneity in the over 45 group, I2 = 87%. There was no significant difference within the groups (*p* = 0.49).

Subgroup analysis based on age indicated that MC intervention was effective in pain reduction (NPRS) in all age groups (Appendix C.3, second row). Patients under 40 years experienced significantly greater pain reduction, MD = −3.11, 95% CI [−3.70, −2.52], I2 = 67%, *p* < 0.01, compared to that in patients over 45 years of age, MD = −1.39, 95% CI [−2.40, −0.39], I2 = 68%, *p* < 0.01. Heterogeneity of both groups were lower than the total heterogeneity of I2 = 79%, with a significant difference between the subgroups (*p* = 0.02).

## 4. Discussion

Both IM and MC interventions resulted in clinically significant pain and disability reduction in CLBP patients according to the ACP definition [32]. IM methods may also be effective in CLBP re-injury risk reduction based on increased trunk extensor endurance [47]. All three intervention groups have often been grouped as one in past syntheses [27,28], which resulted in lower pain or disability reduction as IT intervention was not effective in pain (NPRS) and disability (RMDQ) reduction. Sensitivity analysis resulted in IM and MC interventions being effective in pain and disability reduction. IT was ineffective in reducing pain (NPRS), possibly due to the training loading that imitates some patient-specific spine injury mechanisms [7,8,9,18].

Past intervention studies have indicated that single postural re-education intervention can reduce pain and disability in acute and chronic low back pain patient [64,65]. This result is consistent with the recent meta-analysis on postural re-education for CLBP [66]. MC and IM may be equally effective as both focus on developing the muscular endurance to hold the spine in a neutral position including during limb movement progressions that may have a similar effect with global postural re-education. Most included RCTs comparing both interventions equalised training intensity by having an IM group training duration 30–50% less than that of the MC group, which may cause both groups to have similar outcomes [67,68,69,70].

Inconsistent results in pair-wise meta-analyses with the IT method could be due to the small number of included studies within some pairwise meta-analyses, high risk of bias in some of the included studies, no standard in trunk training frequency and duration, and variability in the recruited patient age group and training duration.

Only three of the included studies used ST in comparing MC and IM [21,34] and only two included studies comparing IT to IM [63,71]. Standardisation and use of a select set of objective outcomes would provide better comparison in future meta-analyses.

Subgroup analysis on CLBP patients trained with IM methods indicated that a longer training duration resulted in further pain reduction, while disability reduction was not significantly different. This indicates that IM intervention may reduce disability earlier than pain. The training duration did not significantly affect pain reduction in MC intervention, indicating that other factors such as clinician skill or difference in training prescription may have an impact that is more significant. Age subgroup analysis indicated that both MC and IM intervention was effective in all age groups, with patients under 40 years experiencing greater pain reduction compared to those over 45 years of age. This could be because older patients require a higher training stimulus to achieve comparable muscular adaptation as that in younger patients [72].

Limitations of the current meta-analysis include a lack of analysis on gender difference, effects of training intensity, and comparison between isolated trunk training and progression with limb movement due to insufficient data. The effect of patient grouping based on specific assessment exceeded the scope of this study. Future research could focus on a single training method in one intervention group to enable a better understanding of the effects of a specific trunk training method on CLBP patient outcomes. In addition, future RCTs should consider incorporating the NPRS, ODI, RMDQ, and ST measurement and follow the CONSORT [73] guidelines to reduce the risk of bias and increase methodological transparency. Future meta-analyses should consider the difference in the recruited patient age demographic and training duration when comparing the different types of CLBP interventions. Other outcome measures and multi-modal interventions may be useful, however, these were excluded to limit the scope of this study.

## 5. Conclusions

Clinicians can prescribe trunk muscle training, focusing on deep abdominal muscle activation (MC method) such as the abdominal draw-in-manoeuvre or isometric trunk muscle activation (IM method) such as the plank for patients with CLBP. Both training approaches can be effective as both methods train the trunk muscle endurance to hold the spine in a neutral position including during active daily living. As the spine in a neutral position is more resilient to tissue injury, CLBP patients trained in the MC and IM methods could gradually experience pain and disability reduction. Trunk muscle training focusing on spine movement (IT method) such as sit-ups may be less effective in pain reduction as it does not train CLBP patients to hold their spine in a neutral position.

Short-term IM training intervention from four to six weeks can result in a pain and disability reduction. CLBP patients with a larger pain score can experience a larger pain reduction with a longer IM intervention of at least eight weeks. Both IM and MC methods may result in larger pain reduction in patients under 40 compared to those over 45. Future CLBP intervention studies should use participants with the same mean age on different groups while future meta-analysis should consider limiting the age range of the included studies’ populations. Further study on the effect of ageing on CLBP training adaptation, and how to adapt training prescription according to CLBP patient age can be investigated in future studies.

## Data Availability

The data in this study can be provided upon reasonable request to the corresponding author.

## Appendix A. Database Search Strategies

CENTRAL search strategy on 24 February 2021	
ID	Search Statement
#1	MeSH descriptor: [Back Pain] explode all trees
#2	dorsalgia
#3	backache
#4	(lumb* or back) next pain
#5	coccyx or coccydynia or spondylosis
#6	MeSH descriptor: [Spine] explode all trees
#7	MeSH descriptor: [Spinal Diseases] explode all trees
#8	lumbago or discitis
#9	disc near herniat*
#10	disk NEAR herniat*
#11	spinal fusion
#12	facet near joint*
#13	MeSH descriptor: [Intervertebral Disc] explode all trees
#14	postlaminectomy
#15	arachnoiditis
#16	failed near back
#17	MeSH descriptor: [Cauda Equina] explode all trees
#18	lumb* near vertebra*
#19	spinal near stenosis
#20	slipped near disc*
#21	slipped NEAR disk*
#22	degenerat* near disc*
#23	degenerat* near disk*
#24	stenosis near spine
#25	stenosis near root
#26	stenosis near spinal
#27	displace* near disc*
#28	displace* near disk*
#29	prolap* near disc*
#30	prolap* near disk*
#31	MeSH descriptor: [Sciatic Neuropathy] explode all trees
#32	sciatic*
#33	back disorder*
#34	#1 OR #2 OR #3 OR #4 OR #5 OR #6 OR #7 OR #8 OR #9 OR #10 OR #11 OR #12 OR #13 OR #14 OR #15 OR #16 OR #17 OR #18 OR #19 OR #20 OR #21 OR #22 OR #23 OR #24 OR #25 OR #26 OR #27 OR #28 OR #29 OR #30 OR #31 OR #32 OR #33
#35	training
#36	endurance
#37	trunk stabil*
#38	lumbar stabil*
#39	exercise
#40	rehab*
#41	core stabil*
#42	transverse abdominis
#43	multifidus
#44	longissimus
#45	extensor
#46	#35 OR #36 OR #37 OR #38 OR #39 OR #40 OR #41 OR #42 OR #43 OR #44 OR #45
#47	#34 AND #46
“trials” tab, 7913 results	

MEDLINE search on 25 February 2021	
ID	Search Statement
#1	clinical trial.mp.
#2	clinical trial.pt.
#3	random:.mp.
#4	1 or 2 or 3
#5	training*.mp.
#6	rehab*.mp.
#7	exercise*.mp.
#8	lumbar stabil*.mp.
#9	trunk stabil*.mp.
#10	core stabil*.mp.
#11	transverse abdominis.mp.
#12	multifidus.mp.
#13	longissimus.mp.
#14	obliques.mp.
#15	5 or 6 or 7 or 8 or 9 or 10 or 11 or 12 or 13 or 14
#16	dorsalgia.mp.
#17	exp Back Pain/
#18	backache.mp.
#19	((lumb* adj pain) or (back adj pain)).mp.
#20	coccyx.mp.
#21	coccydynia.mp.
#22	sciatica.mp.
#23	exp sciatic neuropathy/
#24	spondylosis.mp.
#25	lumbago.mp.
#26	back disorder*.mp.
#27	16 or 17 or 18 or 19 or 20 or 21 or 22 or 23 or 24 or 25 or 26
#28	4 and 15 and 27
#29	limit 28 to humans
2930 results	

PsycINFO search strategy on 25 February 2021	
ID	Search Statement
#1	control:.tw.
#2	random:.tw.
#3	exp treatment/
#4	1 or 2 or 3
#5	back pain/
#6	lumbar spinal cord/
#7	(low adj back adj pain).mp.
#8	(back adj pain).mp.
#9	spinal column/
#10	(lumbar adj2 vertebra*).mp.
#11	coccyx.mp.
#12	sciatica.mp.
#13	lumbago.mp.
#14	dorsalgia.mp.
#15	back disorder*.mp.
#16	“back (anatomy)”/
#17	((disc or disk) adj degenerat*).mp.
#18	((disc or disk) adj herniat*).mp.
#19	((disc or disk) adj prolapse*).mp.
#20	(failed adj back).mp.
#21	5 or 6 or 7 or 8 or 9 or 10 or 11 or 12 or 13 or 14 or 15 or 16 or 17 or 18 or 19 or 20
#22	exp Exercise/ or exercise.mp.
#23	rehabil*.mp.
#24	endurance.mp.
#25	training.mp.
$26	trunk stabil*.mp.
#27	core stabil*.mp.
#28	multifidus.mp.
#29	transverse abdominis.mp.
#30	longissimus.mp.
#31	obliques.mp.
#32	22 or 23 or 24 or 25 or 26 or 27 or 28 or 29 or 30 or 31
#33	4 and 21 and 32
#34	limit 33 to human
1046 results	

EMBASE Search Strategy on 25 February 2021	
ID	Search Statement
#1	random:.tw.
#2	placebo:.mp.
#3	double-blind:.tw.
#4	1 or 2 or 3
#5	dorsalgia.mp.
#6	back pain.mp.
#7	exp BACKACHE/
#8	(lumb* adj pain).mp.
#9	coccyx.mp.
#10	coccydynia.mp.
#11	sciatica.mp.
#12	exp ISCHIALGIA/
#13	spondylosis.mp.
#14	lumbago.mp.
#15	5 or 6 or 7 or 8 or 9 or 10 or 11 or 12 or 13 or 14
#16	exercise*.mp.
#17	rehab*.mp.
#18	lumbar stabil*.mp.
#19	trunk stabil*.mp.
#20	core stabil*.mp.
#21	transverse abdomin*.mp.
#22	multifidus.mp.
#23	longissimus.mp.
#24	obliques.mp.
#25	endurance.mp.
#26	training.mp.
#27	16 or 17 or 18 or 19 or 20 or 21 or 22 or 23 or 24 or 25 or 26
#28	4 and 15 and 27
#29	limit 28 to human
4896 results	

SPORTDiscus search strategy on 25 February 2021	
ID	Search Statement
#1	clinical trial
#2	randomised controlled trial or randomised controlled trial or RCT
#3	1 or 2
#4	low back pain or lumbar spine pain or non-specific low back pain or chronic low back pain
#5	back pain or lumbar pain or spinal pain or backache or lumbago or back injury
#6	4 or 5
#7	trunk control or postural control or core stability or trunk stability
#8	trunk endurance
#9	trunk exercises
#10	core stability exercises
#11	core training
#12	7 or 8 or 9 or 10 or 11
#13	3 and 6 and 12
128 results	

CINAHL search strategy on 25 February 2021	
ID	Search Statement
#1	clinical trial
#2	randomised controlled trial or randomised controlled trial or RCT
#3	1 or 2
#4	low back pain or lumbar spine pain or non-specific low back pain or chronic low back pain
#5	back pain or lumbar pain or spinal pain or backache or lumbago or back injury
#6	4 or 5
#7	trunk control or postural control or core stability or trunk stability
#8	trunk endurance
#9	trunk exercises
#10	core stability exercises
#11	core training
#12	7 or 8 or 9 or 10 or 11
#13	3 and 6 and 12
158 results	

## Appendix B

**Table A1:** Main characteristics of the included randomised controlled trials.

First Author, Year, Country	Group Comparison	Participant Demographics	Inclusion Criteria	Time of Follow Up	Intervention	Adjusted Treatment Effects between Group Mean Change
Aasa, 2015, Sweden	Low-load Motor Control (LMC) i.e., MC (n = 35) High Load Lifting (HLL) i.e., IM (n = 35)	MC: Age 42 ± 11 years, female n = 19 (54%), height 172 ± 10 cm, weight 78 ± 15 kg IM: Age 42 ± 10 years, female n = 20 (57%), height 174 ± 8 cm, weight 74 ± 13 kg	65 subjects, male and female, recruited from clinics. Pain localised to the area of injury/ dysfunction; have a clear, proportionate mechanical nature to aggravating and easing factors; and be intermittent with movement/mechanical provocation	8 weeks	LMC: Individual and home practice, progression to functional movement while maintaining pain free spine position. HLL: group training of deadlift exercise, progressive overload. Both group: 2 months intervention, Week 1–4 have twice/week, week 5–8 have once/week training	NPRS Pre: 43 ± 24 (22, 60), change: MC: −18.5 ± 26.7, IM: −19.0 ± 25.5 Sorensen test, Pre: MC: 75 (63, 87), IM: 87(72, 102); Post: MC: 87 (74, 99), IM: 101 (83, 119) RMDQ: Pre: IM: 7.2 ± 4.3, MC: 7.1 ± 3.9; Post: IM: 3.8 ± 4.0, MC: 3.6 ± 4.2
Akhtar, 2017, Pakistan	Core Stabilisation Exercise i.e., MC (n = 60) Routine Physiotherapy i.e., IT (n = 60)	Mixed gender with unknown ratio MC: Age 46.4 ± 7.4 years, height 162 ± 8 cm, weight 64 ± 10 kg, BMI 24.2 ± 2.4 IM: Age 45.5 ± 6.6 years, height 160 ± 8 cm, weight 63.7 ± 9 kg, BMI 24.8 ± 3	Mechanical CLBP, 20–60 years, M & F, no major spine pathology, surgery, TB, no physio intervention within 6 month	6 weeks	Ultrasound and TENS, twice/week home training, once per week physio training MC: motor control and dynamic surface and functional training Isotonic plus trunk and low limb stretching	MC: 7 discontinued. Isotonic: 5 discontinued NPRS: MC: pre 5.77 ± 1.08, post: 2.69 ± 0.93. Isotonic: pre: 5.40 ± 1.24, post: 3.69 ± 0.79
Areeudomwong, 2016, Thailand	IM (n = 21) Control (n = 21)	IM: Age 35.4 ± 10.3 years, female n = 15 (71.4%), height 162.5 ± 10.5 cm, weight 55.6 ± 7.3 kg Con: Age 36.2 ± 9.9 years, female n = 16 (76.2%), height 163.7 ± 9.4 cm, weight 55.8 ± 8.5 kg	Male or female (n = 42), CLBP, 18–50 years old, at least 2 on NRS Not pregnant, no previous history of spinal surgery, neurological deficits, specific LBP, Cancer, autoimmune disease	4 weeks	IM: 5 times/week, 3 sets of 15 reps of PNF, 30 s rest between set, 60 s rest between posture Control: LBP booklet including home exercise prescription and logbook	NPRS: Pre: IM: 4.08 ± 1.19, Con: 4.15 ± 1.41; Post: 1.46 ± 1.20, Con: 3.08 ± 1.50 RMDQ: Pre: IM: 4.54 ± 0.78, Con: 4.85 ± 1.57; Post: IM: 1.69 ± 0.63, Con: 3.92 ± 1.26
Areeudomwong, 2019, Thailand	MC (n = 15) IM (n = 15) IT (n = 15)	IM: Age 25 ± 8.5 years, female: n = 12 (80%), BMI 22.9 ± 5 MC: Age 24.1 ± 10 years, female: n = 11 (73%), BMI 21.9 ± 4 IM: Age 24.4 ± 10 years, female: n = 11 (73%), BMI 22.6 ± 4	CLBP over 12 weeks, male or female, aged 18–50 No disc herniation, SI joint dysfunction, neurological compromise, surgery, pregnancy, use of other therapies	4 weeks	MC: isolated progressing to functional MC with pressure and EMG biofeedback IM: PNF progressing to combined with upper limb movement IT: Ultrasound plus isolated isotonic training	NPRS: Pre: MC: 4.13 ± 0.92, IM: 4.40 ± 1.40, IT: 4.07 ± 1.28; Post: MC: 1.73 ± 0.96, IM: 2.07 ± 0.88, IT: 3.47 ± 1.55 RMDQ: Pre: MC: 4.53 ± 2.13, IM: 4.60 ± 2.17, IT: 4.47 ± 2.07; Post: MC: 1.47 ± 1.60, IM: 1.93 ± 1.79, IT: 3.93 ± 2.52
Bae, 2018, South Korea	CSE (n = 18) SUE i.e., IT (n = 18)	SUE: Age 32.7 ± 6.1 years, male: female = 9:9, BMI 22.5 ± 2.3 CSE: Age 32.4 ± 10.7 years, male: female 11:7, BMI 22.8 ± 2.2	CLBP with no leg pain >3 months, M & F, NPRS 1–6, aged 20–60, past low back pain includes severe pain severely limiting work and daily activity for >2 days, at least 2×/year No infection, malignancy, inflammatory disease, structural deformity, neurologic sign, abdominal/spinal surgery, pregnancy	4 weeks	CSE: MC progressing to functional movement. IT: machine assisted IT on trunk flexor Both: 30 min session daily exercise, plus 3 session/week	NPRS: Pre: IT: 3.0 ± 1.3, MC: 2.9 ± 0.8; Post: IT: 1.5 ± 1.3, MC: 2.1 ± 0.9 ODI: Pre: IT: 12.8 ± 8.2, MC: 14.2 ± 11.6; Post: IT: 8.0 ± 7.7, MC: 12.0 ± 10.1 RMDQ: Pre: IT: 2.4 ± 1.5, MC: 3.1 ± 2.9; Post: IT: 1.1 ± 1.5, MC: 2.4 ± 2.6
Barradas, 2015, Brazil	MC (n = 5) Con (n = 5)	MC: Age 28.2 ± 3.9 years, BMI 20.8 Con: Age 27.5 ± 3.3 years, BMI 21.1	Female, 20–50 years Exclusion: radiating to lower limbs, acute low back pain, cognitive problem, previous lumbar surgery, cancer, other low back pain intervention	6 weeks	Intervention: Progressive MC training twice/week Con: No detail	NPRS: Pre: MC: 7.2 ± 0.58, Con: 7.80 ± 0.49; Post: MC: 2.60 ± 1.08, Con: 7.30 ± 0.86
Bhadauria, 2017, India	Lumbar stabilisation i.e. MC (n = 12/15) Dynamic strengthening i.e. IT (n = 12/14) Pilates i.e. IM (n = 12/15)	MC: Age 32.8 ± 11 years, male: female = 50:50, BMI 21.8 ± 2.9 IT: Age 36.7 ± 11 years, male: female = 42:58, BMI 24.7 ± 4.6 IM: Age 24.4 ± 11 years, male: female = 42:58, BMI 26 ± 6.2	CLBP > 3 months, male and female 20–60 years, Exclusions: fracture, osteoporosis, degenerative change, spine disc prolapse, bone disorder, arthritis, tumour, radiculopathy, myelopathy, past spine surgery, spine infection, severe psychiatric disorder	6 weeks	All: hot packs, TENS, warm up stretching and cool down totalling 60 min. Lumbar stabilisation: ADIM focused MC training, also have bird dog Dynamic strengthening: IT focused trunk training, has bird dog and hip bridge Pilates: IM trunk muscle contraction in different positions, has hip bridge	NPRS: Pre: MC: 7.17 ± 1.27, IT: 6.67 ± 1.56, IM: 6.42 ± 1.00; Post: MC: 1.17 ± 0.72, IT: 2.00 ± 1.35, IM: 1.33 ± 0.98 ODI: Pre: MC: 39.75 ± 10.11, IT: 37.75 ± 9.27, IM: 28.17 ± 13.55; Post: MC: 6.92 ± 2.47, IT: 23.42 ± 11.01, IM: 8.42 ± 5.14
Cai, 2017, Singapore	LL (n = 28) LE (n = 28) LS (n = 28)	LL: Age 28.9 ± 5.3 years, BMI 21.7 ± 2.4 LE: Age 26.9 ± 6.4 years, BMI 21.8 ± 2.4 LS: Age 26.9 ± 6.4 years, BMI 21.9 ± 2.4	21–45 year old, M & F, 18–25 BMI, CLBP between 3–36 months, running 2–5×/week, 2 km min/session, min 6 month running history, pain intensity between 2–4, no specific spinal pathology, no spine surgery	8 weeks	Both interventions: twice/week LE: progressive isometric extensor training LS: progressive functional MC	NPRS: LE: Pre: 3.44 ± 0.87, Post: 0.76 ± 0.78. LS: Pre: 3.62 ± 1.13, Post: 0.65 ± 0.56
Calatayud, 2020, Spain	IM (n = 42) MC (n = 43)	IM: Age 52 ± 11 years, height 164 ± 10 cm, weight 76 ± 19 kg MC: Age 50 ± 12 years, height 165 ± 7 cm, weight 72 ± 14 kg	NSLBP, aged 18–75, M & F Exclusion: severe somatic condition, psychiatric alteration, neurological disease, spine surgery, participation in other intervention program over past 6 months, exercise contra-indication	8 weeks	IM: Progressive Strength: group based, isolated and functional IM trunk training, 3 times/week MC: 2×/week group training for 3 weeks, followed by home exercise for 5 weeks, ADIM in different position, lumbar and lower limb stretching	NPRS: Pre: MC: 6.3 ± 2, IM: 6.2 ± 2; Post: MC: 5.1 ± 3, IM: 4.3 ± 2 RMDQ: Pre: MC: 10.2 ± 5.52, IM: 7.75 ± 5.08; Post: MC:7.9 ± 5.35, IM: 4.97 ± 4.2 Sorensen Test: Pre: MC: 25.97 ± 29.93, IM: 34.61 ± 28.6; Post: 29.67 ± 28.06, IM: 79 ± 58.19
Cho, 2015, South Korea	Lumbar Stabilisation Exercise i.e., IM (n = 15) Conservative treatment (n = 15)	IM: Age 48.1 ± 6.9 years, height 160.8 ± 6.3 cm, weight 61.9 ± 9.3 kg Con: Age 44 ± 6.7 years, height 163.6 ± 8.2 cm, weight 60.5 ± 12.2 kg	Males and females with CLBP	6 weeks	IM: Side bridge, dead-bug, bird-dog, 3 times/week Con: hot packs (20 min), TENS (15 min), Ultrasound (5 min)	ODI: Pre: IM: 30.1 ± 12.4, Con: 30.4 ± 11.7; Post: IM: 18.4 ± 8.3, Con: 26.2 ± 11.9
Choopani, 2019, Iran	Stabilisation exercise i.e., MC (n = 12) General exercise i.e., IT (n = 12)	MC: Age 48.1 ± 14.5 years, height 161.2 cm, weight 74.9 ± 7.6 kg IT: Age 51.4 ± 6.7 years, height 158.1 cm, weight 77.4 ± 11.6 kg	LBP with or without lower extremities pain >3 months, grade 1 or 2 spondylolisthesis as confirmed with radiology, 20–60 years, M & F, Exclusion: spine surgery, LBP therapy in past 3 months, pregnancy, nervous system disorder, vestibular disorder.	8 weeks	IT: twice/week, daily home exercise, 20 min TENS and hot pack each session Stabilisation group: MC progression to functional movement General group: standard IT trunk training and stretching	NPRS: Pre: MC: 5.16 ± 2.36, IT: 5.5 ± 1.67; Post: MC: 2.07 ± 1.1, 2.73 ± 0.86 ODI: Pre: MC: 48.1 ± 25.5, IT: 29.9 ± 22.47; MC: 44.45 ± 14.36, IT: 25.45 ± 9.3
Cortell-tormo, 2018, Spain	IM (n = 12) Con (n = 12)	IM: Age 35.6 ± 7.9 years, BMI 23.8 ± 2.3 Con: Age 35.6 ± 9.7 years, BMI 24.3 ± 2.4	Women aged 20–55 years, CLBP, no leg pain, pain ≥3 months, ≥3 days/week Exclusion: formal training history, tumour, inflammatory disease, spine or lower limb surgery, spine fracture/deformity, exercise contraindication	12 week	IM: twice/week. 3–4 ppl/group, isolated isometric progressing to functional training Con: no detail	NPRS: Pre: IM: 4 ± 1.8, Con: 4.5 ± 1.6; Post: IM: 1.5 ± 1.5, Con: 4.4 ± 1.4 ODI: Pre: IM: 15.5 ± 8.4, Con: 14 ± 12; Post: IM: 6 ± 6.1, 14.5 ± 11.1 Sorensen: Pre: IM: 81.6 ± 23.6, Con: 66.7 ± 26.7; Post: IM: 136 ± 38.9, Con: 70.1 ± 31.9
Costa 2009, Australia	MC (n = 77) Con (n = 77)	MC: Age 54.6 ± 13 years, female n = 45 (58%), height 165 ± 0.1 cm, weight 74.5 ± 17.5 kg Con: Age 52.8 ± 12.7 years, female n = 48 (62%), height 164 ± 0.1 cm, weight 75.9 ± 15.3 kg	CLBP at least 3 months, 18–80 years, does simple trunk test, not pregnant, not serious spine pathology, no nerve root compromise, no exercise contraindication, M & F	6 weeks	twice/week, 30 min session MC: progressive MC training to functional movement. Placebo: detuned ultrasound at same frequency.	NPRS: MC: Pre: 6.8 ± 2.1, Post: 4.6 ± 2.8. Placebo: Pre: 6.6 ± 2.0, Post: 5.6 ± 2.6 RMDQ: MC: Pre: 13.3 ± 5.0, Post: 9.6 ± 6.5. Placebo: Pre: 13.4 ± 4.9, Post: 11.9 ± 5.9
Farajzadeh, 2017, Iran	IM (n−15) MC (n = 15)	IM: Age 23.8 ± 3.5 years. height 171.8 ± 8 cm, weight 70.5 ± 10.9 kg MC: Age 20.9 ± 1.2 years, height 171.2 ± 7 cm, weight 69.7 ± 12.7 kg	CLBP >3 months, male and female 20–40 years, BMI 20–25, VAS < 4. No pain on lower limb, spinal, abdominal and limb surgery, postural problem due to muscular weakness, limb weakness or pain, neurological defects, cardiovascular disease, professional athlete	6 weeks	IM: 3 times/week, every other day, 30 reps of 10 s of IM trunk training. MC: standard MC exercises, same frequency as IM	NPRS Pre: IM: 2.953 ± 0.485, MC: 2.826 ± 0.654; Post: IM: 2.5 ± 0.49, MC: 2.653 ± 0.787 ODI: Pre: IM: 25.6 ± 9.69, MC: 30.07 ± 11.65; Post: IM: 22.4 ± 9.03, MC: 28 ± 10.16
Franca, 2010, Brazil	SS (n = 15) ST (n = 15)	SS: Age 42.1 ± 8.2 years, BMI 26.4 ± 4.5 SS: Age 41.7 ± 6.4 years, BMI 26.9 ± 3.6	CLBP more than 3 months, no cognitive impairment, no back surgery, spine infection and rheumatologic disorder. No spine exercise within 3 months	6 weeks	twice/week, 30 min sessions. No other exercise. SS: MC training ST: isotonic	NPRS SS: Pre: 5.94 ± 1.56, Post: 0.06 ± 0.16. ST: Pre: 6.49 ± 1.48, Post: 2.89 ± 1.45 ODI: SS: Pre: 17.07 ± 3.99, Post: 1.80 ± 1.26. ST: Pre: 17.27 ± 3.84, Post: 8.40 ± 3.13
Franca, 2019, Brazil	MC (n = 20) TENS (n = 20)	MC: Age 43.1 ± 8.7 years, BMI 26.5 ± 3.7, male: female = 8:12 Con: Age 46.8 ± 7.7 years, BMI 26.5 ± 2.7, male: female = 7:13	18–60 years, with lumbar disc herniation as confirmed via MRI or CT, with or without leg pain no past lumbar surgery, carcinoma, rheumatological disease, stenosis and spondylolisthesis	8 weeks	twice/week, no other physical activity or exercise.	NPRS: Pre: MC:6.4 ± 1.2, Con: 6.3 ± 2.3; Post: MC: 1.5 ± 1.2, Con: 4.8 ± 2.1 ODI (0–45): Pre: MC: 12.3 ± 3.4, Con: 18.0 ± 4.7; Post: MC: 5.1 ± 3.0, Con: 16.2 ± 7.6
Goldby, 2006, United Kingdom	spine stabilisation i.e., MC (n = 84) Manual Therapy (n = 89) Control (n = 40)	MC: Age 43.4 ± 10.7 years, female n = 57 (68%) Con: Age 41.5 ± 13 years, female n = 27 (67.5%)	Mechanical CLBP lasting at least 12 weeks, 18–65 years old, not (pregnant, had back surgery, significant spinal pathology, exercise contra-indication)	10 week	MC: Isolated trunk muscle focused training, patient education Control: patient education	Over 3 months intervention 6 drop out in MC, 4 drop out in MT, 3 drop out in Control NPRS SS: Pre: 4.575 ± 2.754, Post: 2.881 ± 2.814. Control: Pre: 3.76 ± 3.643, Post 3.44 ± 3.643. ODI: SS: Pre: 40.47 ± 15.62, Post: 31.00 ± 17.07. Control: Pre: 33.54 ± 12.21, Post: 28.1 ± 17.34
Gunay 2014, Turkey	Classical strength exercise (CSE) i.e., IT (n = 32) Muscular endurance training (MET) i.e., IM (n = 31)	IT: Age 39.2 ± 7.4 years, BMI 25.2 ± 4.5, female 87.1% IM: Age 40.2 ± 8.0 years, BMI 25.5 ± 3.9, female 81.3%	Age 20–55 years, male and female, over 3 months of CLBP Exclude: spine surgery history, structural deformity, tumour, exercise contraindication	6 week	CSE: trunk, shoulder and hip stretching, dynamic trunk strength training and bird-dog MET: 5 min walk, stretching, short duration, and multiple set and rep isometric hold. Patient posture education, 3 times/week	ODI: Pre MET 32.42 ± 6.49 and CSE 33.59 ± 6.28, p = 0.46. Post MET 18.29 ± 5.21 and CSE 21.09 ± 5.79, p = 0.04 NPRS: Pre MET 5.5 ± 81.36 and CSE 5.4 ± 10.95, p = 0.55. Post MET 2.26 ± 1.12 and CSE 2.56 ± 1.01, p = 0.26 Sorensen: pre MET 49.13 ± 21.92 and CSE 46.6 ± 23.31, p = 0.66. Post MET 98.33 ± 30.11 and CSE 77.03 ± 29.81, p = 0.01
Harts, 2008, Netherlands	High Intensity Training (HIT) i.e., IT (n = 31) Wait List Control (WLC) (n = 21)	HIT: Age: 44 ± 10 years, Con: Age: 41 ± 9 years	18–54 year old male Dutch army over 12 week LBP Exclude: spine surgery within 2 years, severe pain while doing isometric trunk contraction, and nerve root symptoms	8 weeks	HIT: 2 weeks of twice/week, then 6 week of once/week, high intensity dynamic trunk training WLC: wait list	1 missed training from HIT RMDQ (0–24): Pre: HIT: 6.2 ± 4.4, HIT-WLC: −1.4 (−4.0 to 1.1), LIT-WLC: 0.3 (−2.3 to 2.8 for post–pre intervention
Kankaanpaa, 1999, Finland	Active (n = 30) Passive (n = 24)	IT male age: 40.7 ± 8.6 years, BMI: 26.3 ± 2.9. Female age: 38.9 ± 8.2 years, BMI: 25.7 ± 4.3 Con male age: 38.0 ± 6.9 years, BMI: 24.5 ± 3.0. Female age: 40.6 ± 8.1 years, BMI: 25.7 ± 3.2	NSCLBP with moderate functional disability more than 3 months. No previous back surgery or serious spinal pathology. No limb neurological issue. M & F	12 weeks	Active: 24 session, each session 90 min. Group training 4–5 per session. IT trunk training. Passive: thermal and massage therapy 1×/week for 1 month.	2 men and 1 woman drop out of active, 2 men and 5 women drop out of passive therapy Men NPRS: Active: Pre: 5.41 ± 1.96, Post: 3.68 ± 2.88. Passive: Pre: 4.28 ± 2.84, Post: 4.49 ± 2.67 Women NPRS: Active: Pre: 5.61 ± 2.98, Post 2.88 ± 1.88. Passive: Pre: 5.55 ± 3.11, Post 4.18 ± 2.27
Kim 2015, South Korea	CORE (n = 37) Control (n = 37)	CORE: Age 29.7 ± 3.9 years. height 161.3 ± 6.2 cm, weight 56.6 ± 7.1 kg Con: Age 28.6 ± 3.2 years, height 162.8.2 ± 7.8 cm, weight 54.3 ± 7.6 kg	Female office workers with CLBP for over 3 months, 20–40 years, Can move w/o aid Exclude: history of spine or lower limb surgery, spine abnormality, pregnant, no prior exercise intervention	8 weeks	Both: TENS 20 min and hot packs 15 min CORE: 30 min, 5x/week, MC Control: TENS and hot pack	CORE drop out 10 due to pharmacotherapy, surgery and pregnancy. Control drop out 11 due to same reason. NPRS pre: CORE: 5.6 ± 7.9, change at rest CORE: 3.56 ± 0.59, Control 0.58 ± 0.52
Kim, 2020, South Korea	Stretch (n = 25) IM (n = 25) Con (n = 25)	IM: Age 47.0 ± 9.5 years, male: female = 11:11, BMI 23.7 ± 1.5 MC: Age 47.8 ± 8.5 years, male: female = 12:8, BMI 24.0 ± 1.1	CLBP as diagnosed by orthopaedist, pain over 3 months. NPRS ≥ 3. Aged 30–65. Exclude: spinal surgery, ankylosing spondylitis or rheumatoid arthritis, spondylolisthesis or spondylolysis, spine/pelvis fracture, osteoporosis, continuous pain medication, smoking, respiratory/heart disease	6 weeks	IM: hip stretch and strengthening with isometric trunk contraction for 30 min. Con: gentle, sham skin palpitation	NPRS: Pre: IM: 6.12 ± 1.02, Con: 5.85 ± 1.16; Post: IM: 2.37 ± 0.69, Con: 2.92 ± 0.61 ODI: Pre: IM: 56.91 ± 6.92, Con: 58.20 ± 5.27; Post: IM: 30.18 ± 7.66, Con: 36.70 ± 5.12 RMDQ: Pre: IM:11.23 ± 2.62, Con: 11.40 ± 2.28; Post: 3.54 ± 1.59, Con: 5.55 ± 1.82
Kofotolis, 2006, Greece	RST (n = 28) COI (n = 28) Control (n = 30)	RST: age 40.6 ± 6.4 years, BMI 23.7 ± 1.5. COI age: 41.8 ± 7.7 years, BMI 23.7 ± 1.5. Con age: 42.1 ± 8.4 years, BMI: 24.0 ± 1.1	Women. Screening for patients with known mechanical nature of LBP Over 24 weeks CLBP during or after activity No additional physical therapy during intervention	4 weeks	Both groups: 5 times per week, 7–10 min cycling and stretching warm up. RST: isometric contraction, 3 sets of 15 reps COI: alternating eccentric and concentric contraction, 3 sets of 15 reps Con: Active daily living minus exercise	ODI: Pre: RST: 34.8 ± 4.0, COI 36.4 ± 4.4, con 34.2 ± 4.0. Post: RST: 24.6 ± 5.8, COI 24.8 ± 6.4, con 30.8 ± 7.0 NPRS: Pre: RST: 2.2 ± 0.8, COI: 2.3 ± 0.5, Con: 1.9 ± 0.6; Post: RST: 1.4 ± 0.4, COI: 1.4 ± 0.5, Con: 1.6 ± 0.4 Sorensen test score only shown in graphic form
Kofotolis, 2008, Greece	Rhythmic Stabilisation (RS) (n = 23) RS + TENS (n = 23) TENS (n = 23) Con (n = 21)	RST: age 41.0 ± 5.5 years, BMI 24.9 ± 1.2. Con age: 42.2 ± 7.8 years, BMI 23.8 ± 1.7.	92 women, CLBP, pain during or after activity, sitting or climbing stairs, No previous back surgery or serious spinal pathology. No limb neurological issue. No past experience with TENS or RS therapy	4 weeks	RS: Isometric trunk training Placebo: sham TENS	ODI: RS: Pre: 17.1 ± 2.5, Post: 12.6 ± 3.1. Placebo: Pre: 15.7 ± 4.7, Post: 16.7 ± 4.7 NPRS: RS: Pre: 2.1 ± 0.8, Post: 1.6 ± 0.4. Placebo: Pre: 2.1 ± 0.7, Post: 2.0 ± 0.4 Sorensen: RS: Pre: 80.5 ± 6.0, post: 137.0 ± 6.9. Placebo: Pre: 79.0 ± 9.3, Post: 79.0 ± 6.8
Kofotolis, 2016, Greece	Pilates (n = 40) General Strengthening (GS) (n = 40) Control (n = 40)	Con: age 42.7 ± 6.1 years, BMI 24.7 ± 3.8. Pilates age: 41.2 ± 8.5 years, BMI 26.6 ± 3.2. GS 39.1 ± 8.7 years, BMI 23.0 ± 3.7	Female, 25–65 years old, CLBP over 12 weeks, unable to resume daily activity over past 3 weeks. Exclusion: acute low back pain, spinal stenosis or surgery, inflammation affecting spine, fracture, spondylolysis or spondylolisthesis, genetic spinal abnormality, pregnancy, cardiovascular problem, pelvic girdle pain	8 weeks	Both therapy: No additional physiotherapy intervention, 24× 1 h session, 3 times/week, warm up, stretching General Strengthening: Isometric trunk training Pilates: mixed stretch isometric and isotonic Control: No training	37 completed Pilates, 36 completed general strengthening, 28 completed control group. Completed number used in analysis RMDQ: GS: Pre: 12.41 ± 3.69, Post 4.88 ± 1.60. Control: Pre: 11.28 ± 5.40, Post: 10.09 ± 4.55
Koumantakis, 2005, UK	MC (n = 29) IM (n = 26)	MC: 39.2 ± 11.4 years, BMI 26.2 ± 4.2. IM: 35.2 ± 9.7 years, BMI 26.4 ± 3.2	Recurrent CLBP lasting <6 months within past year, confirmed with radiograph or MRI. Onset of current pain >6 weeks, no description on subject gender No past spine surgery, red flags as defined by Clinical Standards Advisory Group (CSAG), spondylolysis, spondylolisthesis	8 week	Both: stretching and stationary bike cycling for 10–15 min. 2×/week class, total 45–60 min/session, total exercise of both group are equalised, home training 30 min, 3 times/week, received patient education booklet MC: progressive ADIM training, 30–45 min, individual followed by home exercise IM: progressive IM training	NPRS: Pre: MC: 2.69 ± 2.06, IM: 4.02 ± 2.46; Post: MC: 1.23 ± 1.37, IM: 2.13 ± 1.73 RMDQ: Pre: MC: 9.2 ± 4.6, IM: 11.3 ± 5.2; Post: MC: 5.1 ± 4.0, IM: 4.7 ± 3.5
Lee, 2016, South Korea	SEG i.e., IM (n = 15) CEG i.e., IM + lower body (n = 15) Con (n = 6)	SEG: 42.7 ± 13.4 years, BMI 24.3 ± 3.1. CEG age 46 ± 8.1 years, BMI 24.1 ± 2.6. Con age 43.3 ± 9.9 years, BMI 27.9 ± 4.4	BMI >23, CLBP, no regular exercise participation in past 6 months. Gender: Not described Subject demographics:	12 weeks	SEG: 2 times/week, 50 min/session Intensity 11–16 Con: No detail	NPRS: Pre: IM: 3.23 ± 1.49, Con: 2.42 ± 0.92; Post: IM: 2.20 ± 1.13, Con: 3.58 ± 1.72 RMDQ: Pre: IM: 3.8 ± 3.7, Con: 0.8 ± 1.2; Post: IM: 1.1 ± 0.9, Con: 1.7 ± 1.6
Majiwala, 2017, India	IM (A) (n = 20) IT (B) (n = 20)	No detail	40 subjects, 20–35 years at physiotherapy department. Patients may be screened for mechanical nature of CLBP, CLBP more than 3 months Exclude: back surgery, injury and trunk training within last 6 months	4 weeks	Both groups: TENS and hot pack A: isometric flexor, extensor and lateral training B: isotonic flexor, extensor and lateral training	NPRS pre-treatment A 7.05 ± 1.317, B 7.3 ± 0.979, p = 0.577. Post treatment A 1.7 ± 0.657, B 1.65 ± 0.745, p = 0.753 Sorensen test pre-treatment A 2.05 ± 0.510, B 2 ± 0.726. Post treatment A 4 ± 0.562, B 3.55 ± 0.510, p = 0.036 ODI pre-treatment A 23.25 ± 2.245, B 27.75 ± 5.884, post-treatment A 7.5 ± 1.573, B 8.8 ± 3.002, p = 0.159
Masharawi 2013, Israel	Intervention (n = 20) Control (n = 20)	MC: 52.5 ± 10.6 years, BMI 27.2 ± 5. Con: 53.6 ± 9.5 years, BMI 26.2 ± 5.5	Female, 45–65 years, with min 12 week CLBP Exclude: obvious structural pathology, overt neurological signs, or joint inflammatory disease	4 week	Intervention: MC (based on paper in reference), bi-weekly intervention. Con: ADL guidance only	NPRS: Pre: MC: 4.0 ± 1.43, Con: 3.91 ± 1.64; Post: MC: 1.68 ± 0.82, Con: 3.88 ± 1.54 RMDQ: Pre: MC: 14.21 ± 5.22, Con: 14.93 ± 5.96, Post: MC: 9.31 ± 5.80, Con: 14.37 ± 5.77
Minobes-Molina, 2020, Spain	TTEP (n = 20) SSEP (n = 20)	TTEP: Age 50.9 ± 11 years, height: 160 ± 10 cm, weight 66.8 ± 9.4 kg, BMI 26.3, SSEP: Age: 50.1 ± 9.8 years, height 160 ± 10 cm, weight 70.9 ± 10 kg, BMI 27.5 ± 3.7	Females aged 18–70, CLBP >6 week confirmed with MRI, CT or radiographic imaging Exclusions: use painkiller, have other spinal disorder, serious co-morbidity, cognitive impairment, exercise contra-indication, recent training participation	6 weeks	Both: First 5 sessions: IR and TENS, no exercise, exercise 3 times/week, TTEP: 30 min IT exercise, 10 movement, 10 repetitions SSEP: 30 min MC exercise, 10 movement, 10 repetitions	NPRS: Pre: IT: 6.4 ± 1.2, MC: 6.5 ± 1.6; Post: IT: 1.9 ± 1.7, MC: 2.2 ± 1.9 RMDQ: Pre: IT: 9.2 ± 3.9, MC: 8.9 ± 4.1, Post: IT: 3.8 ± 3.3, MC: 3.4 ± 2.2
Moon 2013, South Korea	MC (n = 12) IT (n = 12)	IT age: 28.6 ± 4.9 years, male: female = 6:4, height 172.3 ± 6.3 cm, weight 68.2 ± 14.3 kg MC: age: 28.4 ± 5 years, male: female: 8:3, height 171.4 ± 5.1, weight 67.4 ± 12.9 kg	More than 3 months pain No structural or neuropsychological cause, no infectious and systemic disease, fracture, kyphosis, back surgery and other therapy	8 weeks	2 times/week	NPRS Pre: IT: 3.42 ± 1.71, MC: 3.35 ± 1.84, change: IT: 1.41 ± 0.82, MC: 1.67 ± 0.70 ODI Pre: IT: 15.5 ± 4.3, MC: 14.7 ± 2.9 change: IT: 3.6 ± 1.5, MC: 6.1 ± 1.9
Moreira 2019, Brazil	IM (n = 42) Con (n = 42)	No detail on subject demographics	No details on the subjects’ inclusion and exclusion criteria	12 weeks	IM: Functional training Con: No Detail	NPRS: Pre: IM: 6.2 ± 1.3, Con: 6.0 ± 1.7; Post: IM: 2.3 ± 1.9, Con: 5.5 ± 1.8 ODI: Pre: IM: 20.7 ± 10.8, Con: 25.5 ± 11.3; Post: IM: 9.5 ± 6.3, Con: 22.5 ± 10.5 RMDQ: Pre: IM: 6.9 ± 5.0, Con: 8.4 ± 5.3; Post: IM: 2.8 ± 2.6, Con: 7.3 ± 4.7
Steele, 2013, United Kingdom	FullROM (n = 12) Control (n = 9)	FullROM age: 46 ± 12.4 years, BMI: 25.2 ± 3.15 Con age: 41.7 ± 15.1, BMI: 25.94 ± 4.4	Male and female with CLBP for over 12 weeks No contraindication for exercise, acute LBP, pregnancy, disc herniation or neuromuscular complication	12 week	All group: continued medication, avoid other exercise. FullROM: once/week, repetitive 72° lumbar extension with resistance Control: Do nothing	Discontinued: FullROM: 1 for pregnancy, 1 for poor attendance. NPRS change: FullROM −3.03 ± 2.576, Control 0.671 ± 1.489, p < 0.05 ODI: FullROM −18.2 ± 6.63, LimROM −12 ± 5.16, Control −3 ± 6.87, p < 0.05
Shaughnessy, 2004, Ireland	MC (n = 23) Control (n = 22)	Age: MC: 43 ± 9 Con: 46 ± 11 no other demographic details	Inclusion: male and female age 20–60 years, CLBP for min 12 weeks. Exclusion: systemic or structural pathology, inflammatory joint disease, overt neurological signs.	10 weeks	MC: progressive MC to adding limb movement, 2 times/week, Con: Wait list	MC: 3 drop out, Control 1 drop out. ODI: MC: Pre: 37 ± 13, Post: 26 ± 14. Control: Pre: 41 ± 15, Post: 44 ± 16 RMDQ: MC: Pre: 10.0 ± 4.1, Post: 5.1 ± 2.8. Control: Pre: 10.7 ± 5.6, Post: 11.3 ± 5.6
Unsgaard-Tondel, 2010, Norway	Low load MC (n = 36) High load Sling Exercise (n = 36) General Exercise (n = 37)	MC: Age: 40.9 ± 11.5 years, male: female = 7:29, BMI: 24.9 ± 3.1 SE: 43.4 ± 10.2 years, male: female = 13:23, BMI: 24.9 ± 3.1 GE: 36.0 ± 10.3 years, male: female = 13:24, BMI: 24.3 ± 2.8	Male and female, 19–60 years, 3 months min of CLBP, 2–10 score on NPRS (0–10) Exclude: previous back surgery, neurological complication, overweight, pregnancy	8 weeks	MC Exercise (MCE): individualised, 1 on 1, 40 min, n = 36 Sling Exercise (SE): static trunk hold in neutral pose with limb in suspension, 1 on 1, individualised suspended in sling, 40 min, n = 36 Once/weeks, plus home exercise and patient education	MCE: 1 treatment nonadherence, 4 lost on follow up SE: 3 withdrew from study, 3 lost on follow up GE: 7 treatment non-adherence, 3 lost on follow up, 1 withdrew NPRS: MCE: 3.31 ± 1.42, SE 3.61 ± 1.75, GE 3.30 ± 1.74. post: MCE 1.76 ± 1.54, SE 2.34 ± 2.26, GE 2.73 ± 2.32, p = 0.19 ODI: Pre: MCE: 19.44 ± 8.38, SE 22.28 ± 11.22, GE 20.84 ± 9.34. Post: MCE 12.78 ± 7.62, SE 16.18 ± 10.88, GE 17.75 ± 9.63. p = 0.21
You, 2015, Taiwan	Sling exercise (n = 9) Control (n = 7)	Sling: age: 27.6 ± 6.7 years, height 165 ± 7 cm, weight 57.6 ± 12.2 kg. Con: age: 27.6 ± 5.6, height 160 ± 4, weight 57 ± 9.9 kg.	CLBP over 3 months. Exclude: spondylolisthesis and disc herniation	6 weeks	3 times/week, 10 min warm up and 30 min Sling exercise Con: No exercise	2 drop out in sling and 2 drop out in control due to personal reasons NPRS: Control: Pre 3.75 ± 0.87, post 2.38 ± 1.03. Training: Pre 4.29 ± 1.44, post 1.33 ± 1.17 ODI: Control: Pre 19.00 ± 16.20 post 18.50 ± 11.82. Training Pre 14.29 ± 3.90 post 8.86 ± 6.41
Sousa, 2009, Brazil	MC (n = 30) Control (n = 30)	MC: age 45.3 years, male: female = 10:20. Con age 47.5, male: female = 7:23	Male and female, mechanical pain in lumbar spine >3 month Exclude: disc herniation, tumours, infections, osteoarticular inflammatory diseases, fibromyalgia syndrome, chronic obstructive pulmonary disease, or vertebral fracture	8 weeks	Twice/week for, MC with biofeedback Use of 500 mg paracetamol every 6 hrs as necessary on both group Control: No treatment	NPRS: Pre: MC: 4.79 ± 2.73, Control: 5.88 ± 2.99; Post: MC:3.35 ± 2.48, Control: 4.76 ± 2.80 RMDQ: Pre: MC: 9.97 ± 6.00, Control: 12.57 ± 7.30; Post: MC: 5.31 ± 4.79, Control: 8.16 ± 6.20
Oh, 2015, South Korea	Sling SEG (n = 10) Swiss Ball SBEG (n = 10) Control (n = 10)	SEG age 46.2 ± 3.22 years, height 170.1 ± 4.5 cm, weight 71.0 ± 10.5 kg. SBEG age: 46.0 ± 3.4 years, height 172.0 ± 3.2 cm, weight 69.3 ± 9.2 kg Con age: 44.7 ± 2.7 years, height 169.9 ± 4.9 cm, weight 69.2 ± 7.8 kg	Working aged men with normal BMI with CLBP	12 week	SBEG and SEG: isometric trunk holding with limb in various poses. 30 min/session, 5 times/week. Con: active daily living	NPRS: Pre: SBEG: 7.0 ± 0.9, SEG: 7.1 ± 1.6, Con: 6.0 ± 0.9; Post: SBEG: 5.2 ± 1.0, SEG: 4.5 ± 1.3, Con: 5.4 ± 0.9 ODI (out of 100): Pre: SBEG: 28.0 ± 6.3, SEG: 32.4 ± 6.7, Con: 18.9 ± 7.0. Post: 13.8 ± 6.3, SEG: 10.4 ± 4.2, Con: 14.5 ± 4.7
Yi, 2008, South Korea	IM (n = 20) IT (n = 20) IM + IT (n = 20)	IM age 51.6 ± 9.4 years, male: female = 2:12. IT age 50.2 ± 10.5 years, male: female = 2:15	CLBP >6 months, 20–60 years old Exclude: lower limb radiating pain, pregnant, artificial joint	8 weeks	No details	ODI: Pre: IM: 27.9 ± 15.5, IT: 29.5 ± 12.6; Post: IM: 14.1 ± 8.9, IT: 16.6 ± 11.7 NPRS: Pre: IM: 5.01 ± 2.92, IT: 5.39 ± 2.90; Post: IM: 1.46 ± 1.45, IT: 1.75 ± 1.63
Shamsi 2015, Iran	MC (n = 19) IM (n = 20)	MC: age 38.5 ± 11.9 years, male: female = 6:13, height 166.7 ± 8.6 cm, weight 68.9 ± 15.7 kg IM: age 47.7 ± 10.4 years, male: female = 6:14, height 163.7 ± 8.3 cm, weight 73.1 ± 8.9 kg	Male and female with CLBP >3 months, aged 18–60 years, NPRS 3–6 Exclude: lower limb pathology or anomaly, inflammatory diseases, osteoporosis, arthritis, bone disease	6 weeks	3 times/week intensity equalised	NPRS: Pre: MC: 5.24 ± 0.92, IM: 5.30 ± 0.92; Post: MC: 1.59 ± 1.24, IM: 1.49 ± 1.41 ODI: Pre: MC: 51.1 ± 12.7, IM: 49.8 ± 10.8; Post: MC: 33.3 ± 11.0, IM: 37.4 ± 11.1
Segal-Snir 2016, Israel	Intervention (n = 25) Control (n = 20)	IT: age 57.2 ± 8.4 years, BMI 27.6 ± 4.5 Con: age 54.7 ± 6.5 years, BMI 28.5 ± 5.5	45 female aged 40–70 years, CLBP >12 weeks. Exclude: systemic or structural pathology, inflammatory joint disease or neurological signs.		Control: Wait list Intervention: 40 min/session, 2 times/week, 4 week duration, 10 person/group max, 5 position IT exercises.	NPRS: Pre: IT: 7 ± 2.3, Con: 8 ± 1.4; Post: IT: 7 ± 3, Con: 7 ± 1.9 RMDQ: Pre: IT: 13 ± 6.2, Con: 14 ± 6.3; Post: IT: 11 ± 6.4, Con: 14 ± 6.3
Noormohammadpour 2018, Iran	Intervention (n = 18) Control (n = 18)	MC age 43.3 ± 7.5 years, BMI 24.0 ± 1.7 Con age 41.3 ± 6.4 years, BMI 24.3 ± 2.1	Female nurses with CLBP >3 months in past 6 months, aged 18–55, Exclude: spine/abdominal trauma/surgery, systemic disease, spine deformity, abdominal wall hernia, participation in core stability intervention	8 weeks	Intervention: 2 floor and 2 swiss ball exercises, ADIM focused, progressing to functional movement, home exercise 3 times/day for 10 reps Con: Wait list	NRPS: Pre: MC: 5.18 ± 2.41, Con: 4.42 ± 2.65; Post: MC: 1.24 ± 1.35, Con: 3.94 ± 2.09 RMDQ: Pre: MC: 7.8 ± 3.4, Con: 9.5 ± 4.9; Post: MC: 1.7 ± 2.4, Con: 7.9 ± 3.3
Rathod 2015, Pakistan	MC (n = 20) IM (n = 20)	No demographic data	Male and female clerks with CLBP >3 months, age 30–45 years Exclude: lumbar spondylolysis, spondylolisthesis, acute disc prolapse, neurological disorders, other muscoskeletal disorders, hypertension and ischaemic heart disease	4 week	Both: 20 min ultrasound first 10 days, MC: Progressive trunk training with ADIM with help of biofeedback unit IM: IM trunk training, 5–10 s hold, 5–20 reps	NPRS: Pre: MC: 7.30 ± 0.97, IM: 6.80 ± 1.36; Post: MC: 0.25 ± 0.78, IM: 1.20 ± 1.10 ODI (0–100): Pre: MC: 45.76 ± 7.81, IM: 46.67 ± 6.55; Post: MC: 8.21 ± 7.94, IM: 24.86 ± 7.20
Shamsi 2016, Iran	MC (n = 24) IM (n = 24)	MC age 39.2 ± 11.7 years, male: female = 7:15, height 166.4 ± 9.1 cm, weight 70.1 ± 15.1 kg IM age 47.9 ± 10.2 years, male: female = 6:15, height 163.7 ± 8.1 cm, weight 74.3 ± 10.5 kg	CLBP >3 months, VAS 3–6, 18–60 years. Exclude: lumbar radicular pain, lower limb pathology or anomaly	6 weeks	Both: stretching 8 min, stationary cycling 5 min, progressive exercise, group training, 3× per week MC: 20 min trunk training, focused on ADIM IM: 14 min trunk training, focused on extensor and flexor muscles	NPRS: Pre: MC: 5.14 ± 0.98, IM: 5.07 ± 1.13; Post: MC: 1.51 ± 1.18, IM: 1.51 ± 1.38 ODI (0–100): Pre: MC: 50.5 ± 12.1, IM: 50.7 ± 11.3; Post: MC: 32.8 ± 10.5, IM: 37.6 ± 10.9 Sorensen: Pre: MC: 70.6 ± 57.1, IM: 80.9 ± 48.6; Post: MC: 117.2 ± 60.2, IM: 113.2 ± 52.0
Shamsi, 2020, Iran	Core Stability i.e., MC (n = 28) General Exercise i.e., IM (n = 28)	MC age 38.9 ± 12.2 years, male: female = 11:16, height 167.6 ± 8.8 cm, weight 71.9 ± 14.2 kg IM age 47.0 ± 9.9 years, male: female = 7:17, height 164.0 ± 9.1 cm, weight 74.2 ± 10.7	Male and female, CLBP based on imaging and pain provocation, duration >3 months, NPRS = 3–6, aged 18–60, M & F Exclude: spine and lower limb pathology or anomaly	6 week	Both: 8 min stretching and 5 min stationary cycling, 3×/week Core Stability: MC training, focused on ADIM and isolated deep muscle contraction General Exercise: Isolated floor IM trunk training based on description of (Shamsi 2015)	ODI (0–100): Pre: MC: 50.55 ± 12.08, IM: 50.67 ± 10.41; Post: MC: 32.77 ± 11.0, IM: 37.62 ± 10.87 NPRS: Pre: MC: 5.136 ± 0.902, IM: 5.286 ± 0.902; Post: MC: 1.509 ± 1.24, IM: 1.510 ± 1.380
Cruz-Diaz 2017, Spain	Mat Pilates group i.e., MC (n = 34) Con (n = 30)	MC age 36.9 ± 12.5 years, male: female = 11:23, height 167.6 ± 8.8 cm, weight 71.9 ± 14.2 kg Con age 36.3 ± 10.7 years, male: female = 13:21, height 164.0 ± 9.1 cm, weight 74.2 ± 10.7 kg	CLBP >12 weeks, age 18–50, NPRS 3–7 Exclude: radiculopathy, fracture, stenosis, tumour, Pilates or physical therapy training over past 6 months. Not pregnant.	12 weeks	Pilates Mat Group: warm up, training and cool down. Focus on pelvic tilt and ADIM, joint mobility drills. Training in group of 4. Control: Not described	NPRS: Pre: MC: 4.64 ± 1.22, Con: 4.84 ± 1.04; Post: MC: 2.1 ± 1.36, Con: 4.96 ± 1.31 RMDQ: Pre: MC: 11.38 ± 5.02, Con: 10.50 ± 4.89; Post: MC: 6.35 ± 5.3, Con: 10.41 ± 5.6
Kim 2017, South Korea	PNF i.e., IM (n = 15) Con (n = 15)	IM age 39.8 ± 5.5 years, male: female = 8:7, height 168.7 ± 7.3 cm, weight 67.6 ± 9.5 kg. Con age 39.4 ± 5.7 years, male: female 9:6, height 168.7 ± 8.0 cm, weight 67.1 ± 9.7 kg.	CLBP >12 weeks, age 30–40 years, no exercise/mental problem, neurologic sensation, muscular paralysis.	6 weeks	PNF: isometric holding in different position, resisting force in different direction while maintaining normal breathing. Control: no description 5 times/weeks	NPRS: Pre: IM: 6.6 ± 1.12, Con: 6.5 ± 1.11; Post: IM: 2.4 ± 0.54, Con: 4.6 ± 0.90. ODI: Pre: IM: 33.3 ± 6.8, Con: 33.5 ± 4.8; Post: IM: 21.3 ± 3.4, Con: 28.9 ± 4.6
